# PyGeNN: A Python Library for GPU-Enhanced Neural Networks

**DOI:** 10.3389/fninf.2021.659005

**Published:** 2021-04-22

**Authors:** James C. Knight, Anton Komissarov, Thomas Nowotny

**Affiliations:** ^1^Centre for Computational Neuroscience and Robotics, School of Engineering and Informatics, University of Sussex, Brighton, United Kingdom; ^2^Bernstein Center for Computational Neuroscience Berlin, Berlin, Germany; ^3^Department of Engineering and Computer Science, Technische Universität Berlin, Berlin, Germany

**Keywords:** GPU, high-performance computing, parallel computing, benchmarking, computational neuroscience, spiking neural networks, python

## Abstract

More than half of the Top 10 supercomputing sites worldwide use GPU accelerators and they are becoming ubiquitous in workstations and edge computing devices. GeNN is a C++ library for generating efficient spiking neural network simulation code for GPUs. However, until now, the full flexibility of GeNN could only be harnessed by writing model descriptions and simulation code in C++. Here we present PyGeNN, a Python package which exposes all of GeNN's functionality to Python with minimal overhead. This provides an alternative, arguably more user-friendly, way of using GeNN and allows modelers to use GeNN within the growing Python-based machine learning and computational neuroscience ecosystems. In addition, we demonstrate that, in both Python and C++ GeNN simulations, the overheads of recording spiking data can strongly affect runtimes and show how a new spike recording system can reduce these overheads by up to 10×. Using the new recording system, we demonstrate that by using PyGeNN on a modern GPU, we can simulate a full-scale model of a cortical column faster even than real-time neuromorphic systems. Finally, we show that long simulations of a smaller model with complex stimuli and a custom three-factor learning rule defined in PyGeNN can be simulated almost two orders of magnitude faster than real-time.

## 1. Introduction

A wide range of spiking neural network (SNN) simulators are available, each with their own application domains. NEST (Gewaltig and Diesmann, 2007) is widely used for large-scale point neuron simulations on distributed computing systems; NEURON (Carnevale and Hines, 2006) and Arbor (Akar et al., 2019) specialize in the simulation of complex multi-compartmental models; NeuroKernel (Givon and Lazar, 2016) is focused on emulating fly brain circuits using Graphics Processing Units (GPUs); and CARLsim (Chou et al., 2018), ANNarchy (Vitay et al., 2015), Spice (Bautembach et al., 2021), NeuronGPU (Golosio et al., 2021), and GeNN (Yavuz et al., 2016) use GPUs to accelerate point neuron models. For performance reasons, many of these simulators are written in C++ and, especially amongst the older simulators, users describe their models either using a Domain-Specific Language (DSL) or directly in C++. For programming language purists, fully custom DSLs such as the HOC network description language in NEURON (Carnevale and Hines, 2006) or the NestML (Plotnikov et al., 2016) neuron modeling language may be elegant solutions and, for simulator developers, using C++ directly and not having to add bindings to another language is convenient. However, both choices act as a barrier to potential users. Therefore, with both the computational neuroscience and machine learning communities gradually coalescing toward a Python-based ecosystem with a wealth of mature libraries for scientific computing (Hunter, 2007; Millman and Aivazis, 2011; Van Der Walt et al., 2011), exposing spiking neural network simulators to Python with minimal domain specific modifications seems like a pragmatic choice. NEST (Eppler et al., 2009), NEURON (Hines et al., 2009), and CARLsim (Balaji et al., 2020) have all taken this route and now all offer Python interfaces. Furthermore, newer simulators such as Arbor and Brian2 (Stimberg et al., 2019) have been designed from the ground up with a Python interface.

Our GeNN simulator can already be used as a backend for the Python-based Brian2 simulator (Stimberg et al., 2019) using the Brian2GeNN interface (Stimberg et al., 2020) which modifies the C++ backend “cpp_standalone” of Brian 2 to generate C++ input files for GeNN. As for cpp_standalone, initialization of simulations is mostly done in C++ on the CPU and recording data is saved into binary files and re-imported into Python using Brian 2's native methods. While we have recently demonstrated some very competitive performance results (Knight and Nowotny, 2018, 2020) using GeNN in C++, and through the Brian2GeNN interface (Stimberg et al., 2020), GeNN could so far not be used directly from Python and it is not possible to expose all of GeNN's unique features through the Brian2 API. Specifically, GeNN not only allows users to easily define their own neuron and synapse models but, also “snippets” for offloading the potentially costly initialization of model parameters and connectivity onto the GPU. Additionally, GeNN provides a lot of freedom for users to integrate their own code into the simulation loop. In this paper we describe the implementation of PyGeNN—a Python package which aims to expose the full range of GeNN functionality with minimal performance overheads. Unlike in the majority of other SNN simulators PyGeNN allows defining bespoke neuron and synapse models directly from Python without requiring users to extend the underling C++ code. Below, we demonstrate the flexibility and performance of PyGeNN in two scenarios where minimizing performance overheads is particularly critical.

- In a simulation of a large, highly-connected model of a cortical microcircuit (Potjans and Diesmann, 2014) with small simulation timesteps. Here the cost of copying spike data off the GPU from a large number of neurons every timestep can become a bottleneck.
- In a simulation of a much smaller model of Pavlovian conditioning (Izhikevich, 2007) where learning occurs over 1 h of biological time and stimuli are delivered—following a complex scheme—throughout the simulation. Here any overheads are multiplied by a large number of timesteps and copying stimuli to the GPU can become a bottleneck.

Using the facilities provided by PyGeNN, we show that both scenarios can be simulated from Python with only minimal overheads over a pure C++ implementation.

## 2. Materials and Methods

### 2.1. GeNN

GeNN (Yavuz et al., 2016) is a library for generating CUDA (NVIDIA et al., 2020) code for the simulation of spiking neural network models. GeNN handles much of the complexity of using CUDA directly and automatically performs device-specific optimizations so as to maximize performance. GeNN consists of a main library—implementing the API used to define models as well as the generic parts of the code generator—and an additional library for each backend (currently there is a reference C++ backend for generating CPU code and a CUDA backend. An OpenCL backend is under development). Users describe their model by implementing a modelDefinition function within a C++ file. For example, a model consisting of four Izhikevich neurons with heterogeneous parameters, driven by a constant input current might be defined as follows:


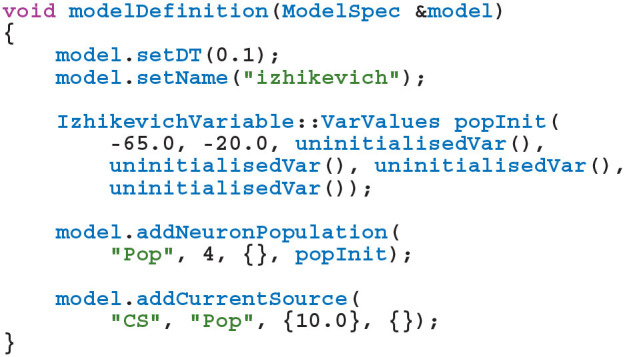


The *genn-buildmodel* command line tool is then used to compile this file; link it against the main GeNN library and the desired backend library; and finally run the resultant executable to generate the source code required to build a simulation dynamic library (a .dll file on Windows or a .so file on Linux and Mac). This dynamic library can then either be linked against a simulation loop provided by the user or dynamically loaded by the user's simulation code. To demonstrate this latter approach, the following example uses the SharedLibraryModel helper class supplied with GeNN to dynamically load the previously defined model, initialize the heterogenous neuron parameters and print each neuron's membrane voltage every timestep:


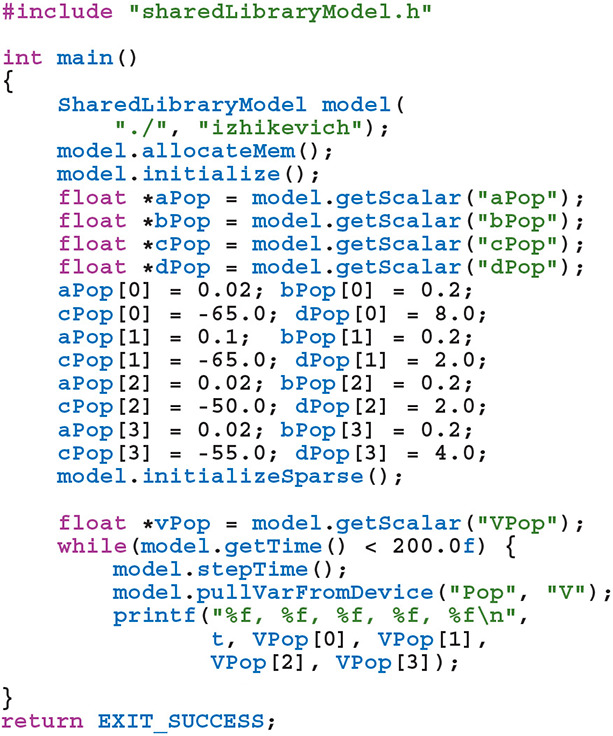


### 2.2. SWIG

In order to use GeNN from Python, both the model creation API and the SharedLibraryModel functionality need to be “wrapped” so they can be called from Python. While this is possible using the API built into Python itself, wrapper functions would need to be manually implemented for each GeNN function to be exposed which would result in a lot of maintenance overhead. Instead, we chose to use SWIG (Beazley, 1996) to automatically generate wrapper functions and classes. SWIG generates Python modules based on special interface files which can directly include C++ code as well as special “directives” which control SWIG. For example, the following SWIG interface file would wrap the C++ code in test.h in a Python module called test_module within a Python package called test_package:


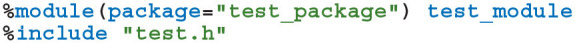


The %module directive sets the name of the generated module and the package it will be located in and the %include directive parses and automatically generates wrapper functions for the C++ header file. We use SWIG in this manner to wrap both the model building and SharedLibraryModel APIs described in section 2.1. However, key parts of GeNN's API such as the ModelSpec::addNeuronPopulation method employed in section 2.1, rely on C++ templates which are not directly translatable to Python. Instead, valid template instantiations need to be given a unique name in Python using the %template SWIG directive:


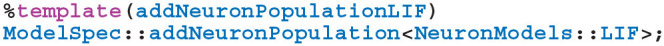


Having to manually add these directives whenever a model is added to GeNN would be exactly the sort of maintenance overhead we were trying to avoid by using SWIG. Therefore, when building the Python wrapper, we instead search the GeNN header files for the macros used to declare models in C++ and automatically generate SWIG %template directives.

As previously discussed, a key feature of GeNN is the ease with which it allows users to define their own neuron and synapse models as well as “snippets” defining how variables and connectivity should be initialized. Beneath the syntactic sugar described in our previous work (Knight and Nowotny, 2018), new models are defined by simply writing a new C++ class derived from, for example, the NeuronModels::Base class. Being able to define such classes from Python was a key requirement of PyGeNN. However, to support this, GeNN's C++ code generator would need to be able to call through to the methods in the Python class used by the user to implement a model. SWIG makes this easy by generating all of the boilerplate code required to make C++ classes inheritable from Python using a single SWIG “director” directive:


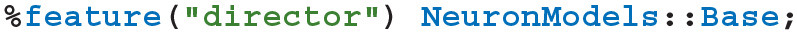


### 2.3. PyGeNN

While GeNN *could* be used from Python via the wrapper generated using SWIG, the resultant code would be unpleasant to use directly. For example, rather than being able to specify neuron parameters using native Python types such as lists or dictionaries, one would have to use a wrapped type such as DoubleVector([0.25, 10.0, 0.0, 0.0, 20.0, 2.0, 0.5]). Therefore, in order to provide a more user-friendly and pythonic interface, we have built PyGeNN on top of the wrapper generated by SWIG. PyGeNN combines the separate model building and simulation stages of building a GeNN model in C++ into a single API, likely to be more familiar to users of existing Python-based model description languages such as PyNEST (Eppler et al., 2009) or PyNN (Davison et al., 2008). By combining the two stages together, PyGeNN can provide a unified dictionary-based API for initializing homogeneous and heterogeneous parameters as shown in this re-implementation of the previous example:


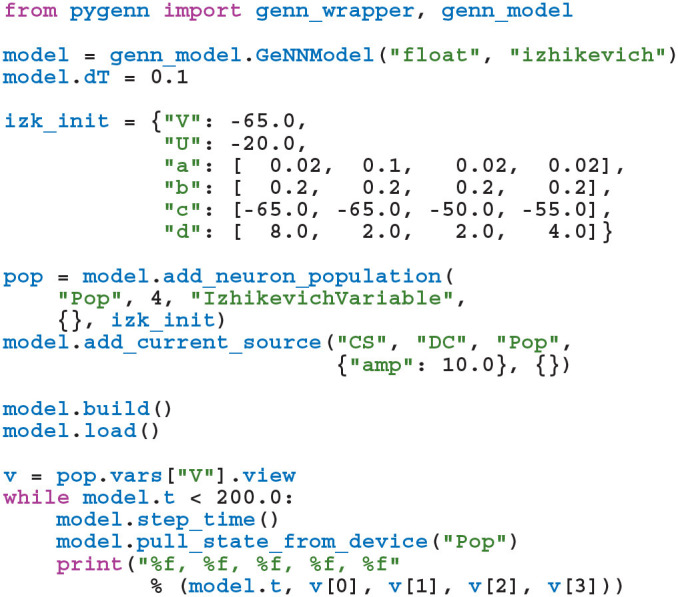


Initialization of variables with homogeneous values—such as the neurons' membrane potential—is performed by initialization kernels generated by GeNN and the initial values of variables with heterogeneous values—such as the a, b, and c parameters—are copied to the GPU by PyGeNN after the model is loaded. While the PyGeNN API is more pythonic and, hopefully, more user-friendly than the C++ interface, it still provides users with the same low-level control over the simulation. Furthermore, by using SWIG's numpy (Van Der Walt et al., 2011) interface, the host memory allocated by GeNN can be accessed directly from Python using the pop.vars["V"].view syntax meaning that no potentially expensive additional copying of data is required.

As illustrated in the previously-defined model, for convenience, PyGeNN allows users to access GeNN's built-in models. However, one of PyGeNN's most powerful features is that it enables users to easily define their own neuron and synapse models from within Python. For example, an Izhikevich neuron model (Izhikevich, 2003) can be defined using the create_custom_neuron_class helper function which provides some syntactic sugar over directly inheriting from the SWIG director class:


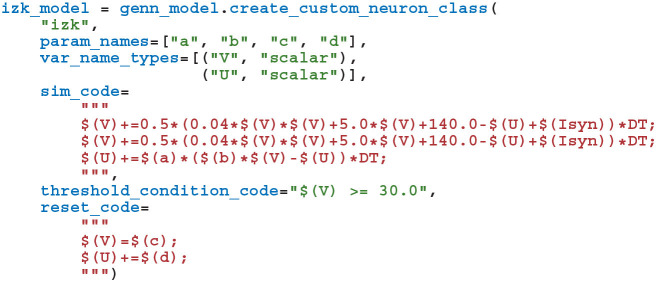


The param_names list defines the real-valued parameters that are constant across the whole population of neurons and the var_name_types list defines the model state variables and their type (the scalar type is an alias for either single or double-precision floating point, depending on the precision passed to the GeNNModel constructor). The behavior of the model is then defined using a number of code strings. Unlike in tools like Brian 2 (Stimberg et al., 2019), these code strings are specified in a C-like language rather than using differential equations. This language provides standard C control flow statements as well as the transcendental functions from the standard maths library. Additionally, variables provided by GeNN such as the membrane voltage in the model above can be accessed using the $(V) syntax and functions provided by GeNN can be called using the $(F, 1, 2) syntax (where F is a 2 argument function). Using C-like code strings allows expert users to choose their own solver for models described in terms of differential equations and to programatically define models such as spike sources. For example, in the model presented above, we chose to implement the neuron using the idiosyncratic forward Euler integration scheme employed by Izhikevich (2003). Finally, the threshold_condition_code expression defines *when* the neuron will spike whereas the reset_code code string defines how the state variables should be reset after a spike.

### 2.4. Spike Recording System

Internally, GeNN stores the spikes emitted by a neuron population during one simulation timestep in an array containing the indices of the neurons that spiked alongside a counter of how many spikes have been emitted overall. Previously, recording spikes in GeNN was very similar to the recording of voltages shown in the previous example code—the array of neuron indices was simply copied from the GPU to the CPU every timestep. However, especially when simulating models with a small simulation timestep, such frequent synchronization between the CPU and GPU is costly—especially if a slower, interpreted language such as Python is involved. Furthermore, biological neurons typically spike at a low rate (in the cortex, the average firing rate is only around 3 Hz; Buzsáki and Mizuseki, 2014) meaning that the amount of spike data transferred every timestep is typically very small. One solution to these inefficiencies is to store many timesteps worth of spike data on the GPU and use more infrequent, larger transfers to copy it to the CPU.

When a model includes delays, the array of indices and the counter used to store spikes internally are duplicated for each delay “slot.” Additional delay slots could be artificially added to the neuron population so that this data structure could be re-used to also store spike data for subsequent recording. However, the array containing the indices has memory allocated for all neurons to handle the worst case where all neurons in the population fire in the same time step. Therefore, while this data structure is ideal for efficient spike propagation, using it to store many timesteps worth of spikes would be very wasteful of memory. At low firing rates, the most memory efficient solution would be to simply store the indices of neurons which spiked each timestep, for example in a data structure similar to a Yale sparse matrix with each “row” representing a timestep (Eisenstat et al., 1977). However, not only would the efficiency of this approach rely on GeNN *only* being used for models with biologically-plausible firing rates, but the amount of memory required to store the spikes for a given number of timesteps could not be determined ahead of time. Therefore, either GeNN or the user would need to regularly check the level of usage to determine whether the buffer was exhausted, leading to exactly the type of host-synchronization overheads the spike recording system is designed to alleviate. Instead, we represent the spikes emitted by a population of *N* neurons in a single simulation timestep as a *N*bit bitfield where a “1” represents a spike and a “0” the absence of one. Spiking data over multiple timesteps is then represented by a circular buffer of these bitfields. While at very low firing rates, this approach uses more memory than storing the indices of the neurons which spiked, it still allows the spiking output of relatively large models, running for many timesteps to be stored in a small amount of memory. For example, the spiking output of a model with 100 × 10^3^ neurons running for 10 × 10^3^ simulation timesteps, required <120 MB—a small fraction of the memory on a modern GPU. While efficiently handling spikes stored in a bitfield is a little trickier than working with a list of neuron indices, GeNN provides an efficient C++ helper function for saving the spikes stored in a bitfield to a text file and a numpy-based method for decoding them in PyGeNN.

### 2.5. Cortical Microcircuit Model

**Figure 1:**
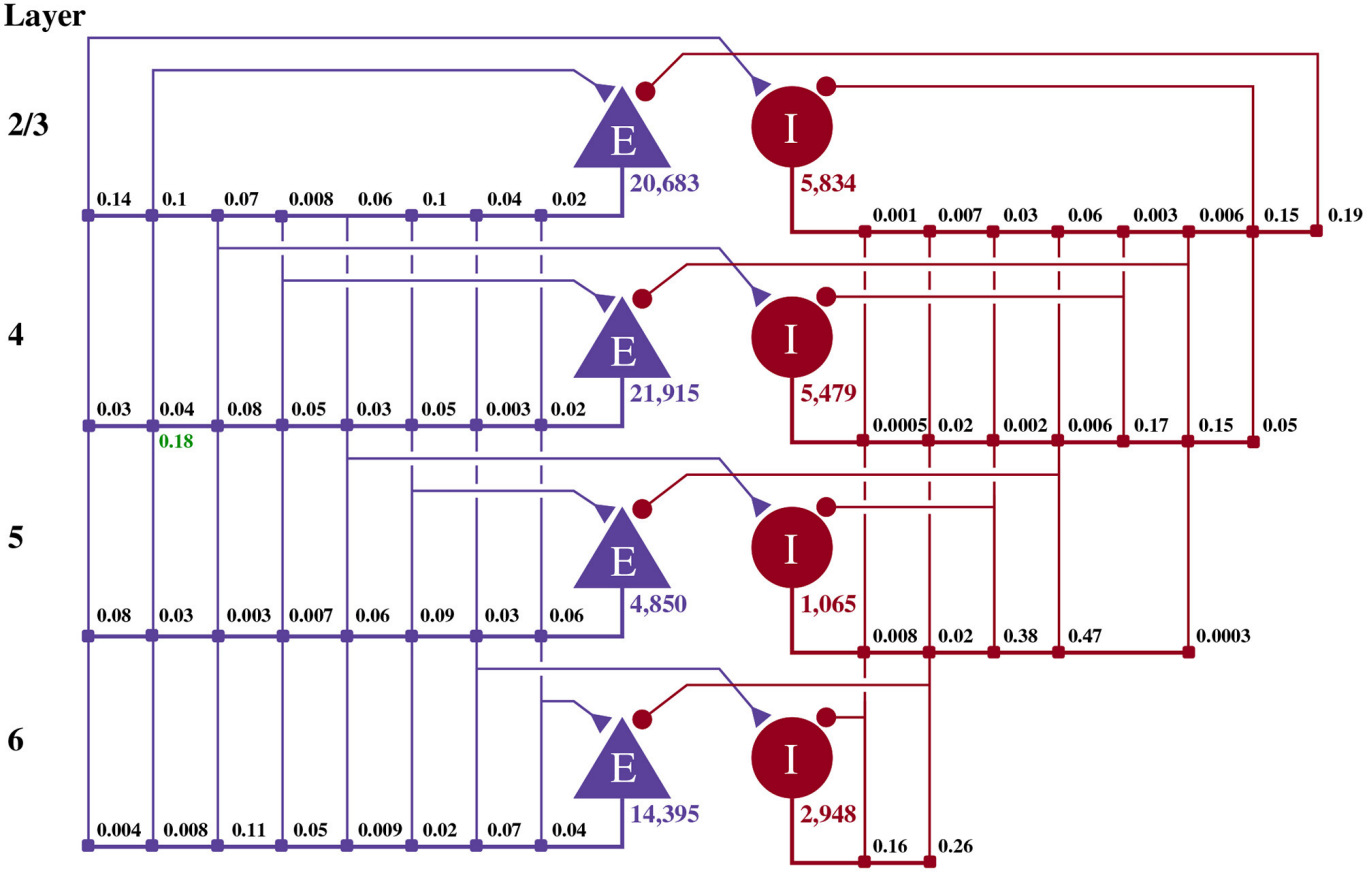
Illustration of the microcircuit model. Blue triangles represent excitatory populations, red circles represent inhibitory populations, and the number beneath each symbol shows the number of neurons in each population. Connection probabilities are shown in small bold numbers at the appropriate point in the connection matrix. All excitatory synaptic weights are normally distributed with a mean of 0.0878 nA (unless otherwise indicated in green) and a standard deviation of 0.0878 nA. All inhibitory synaptic weights are normally distributed with a mean of 0.3512 nA and a standard deviation of 0.03512 nA.

Potjans and Diesmann (2014) developed the cortical microcircuit model of 1 mm2 of early-sensory cortex illustrated in Figure 1. The model consists of 77,169 LIF neurons, divided into separate populations representing the excitatory and inhibitory population in each of four cortical layers (2/3, 4, 5, and 6). The membrane voltage *V*_*i*_ of each neuron *i* is modeled as:

(1) $$\begin{array}{l} \tau_{\text{m}}\frac{dV_{i}}{dt}=\left(V_{\text{rest}}-V_{i}\right)+R_{\text{m}}\left(I_{{\text{syn}}_{i}}+I_{{\text{ext}}_{i}}\right), \end{array}$$

where τ_m_ = 10 ms and *R*_m_ = 40MΩ represent the time constant and resistance of the neuron's cell membrane, *V*_rest_ = −65 mV defines the resting potential, *I*_syn_*i*__ represents the synaptic input current and *I*_ext_*i*__ represents an external input current. When the membrane voltage crosses a threshold *V*_th_ = −50 mV a spike is emitted, the membrane voltage is reset to *V*_rest_ and updating of *V* is suspended for a refractory period τ_ref_ = 2 ms. Neurons in each population are connected randomly with numbers of synapses derived from an extensive review of the anatomical literature. These synapses are current-based, i.e., presynaptic spikes lead to exponentially-decaying input currents *I*_syn_*i*__

(2) $$\begin{array}{l} \tau_{\text{syn}}\frac{dI_{{\text{syn}}_{i}}}{dt}=-I_{{\text{syn}}_{i}}+\overset{n}{\underset{j=0}{\sum }}w_{ij}\underset{t_{j}}{\sum }\delta \left(t-t_{j}\right), \end{array}$$

where τ_syn_ = 0.5 ms represents the synaptic time constant, *w*_*ij*_ represents the synaptic weight and *t*_*j*_ are the arrival times of incoming spikes from *n* presynaptic neurons. Within each synaptic projection, all synaptic strengths and transmission delays are normally distributed using the parameters presented in Potjans and Diesmann (2014, Table 5) and, in total, the model has approximately 0.3 × 10^9^ synapses. As well as receiving synaptic input, each neuron in the network also receives an independent Poisson input current, representing input from neighboring not explicitly modeled cortical regions. The Poisson input is delivered to each neuron via *I*_ext_*i*__ with

(3) $$\begin{array}{l} \tau_{\text{syn}}\frac{dI_{{\text{ext}}_{i}}}{dt}=-I_{{\text{ext}}_{i}}+w_{\text{ext}}\text{Poisson}\left(\nu_{\text{ext}}\Delta t\right), \end{array}$$

where ν_ext_ represents the mean input rate and *w*_ext_ represents the weight. The ordinary differential Equations (1), (2), and (3) are solved with an exponential Euler algorithm. For a full description of the model parameters, please refer to Potjans and Diesmann (2014, Tables 4, 5) and for a description of the strategies used by GeNN to parallelize the initialization and subsequent simulation of this network, please refer to Knight and Nowotny (2018, section 2.3). This model requires simulation using a relatively small timestep of 0.1 ms, making the overheads of copying spikes from the GPU every timestep particularly problematic.

### 2.6. Pavlovian Conditioning Model

The cortical microcircuit model described in the previous section is ideal for exploring the performance of short simulations of relatively large models. However, the performance of longer simulations of smaller models is equally vital. Such models can be particularly troublesome for GPU simulation as, not only might they not offer enough parallelism to fully occupy the device but, each timestep can be simulated so quickly that the overheads of launching kernels etc can dominate. Additional overheads can be incurred when models require injecting external stimuli throughout the simulation. Longer simulations are particularly useful when exploring synaptic plasticity so, to explore the performance of PyGeNN in this scenario, we simulate a model of Pavlovian conditioning using a three-factor Spike-Timing-Dependent Plasticity (STDP) learning rule (Izhikevich, 2007).

#### 2.6.1. Neuron Model

**Figure 2:**
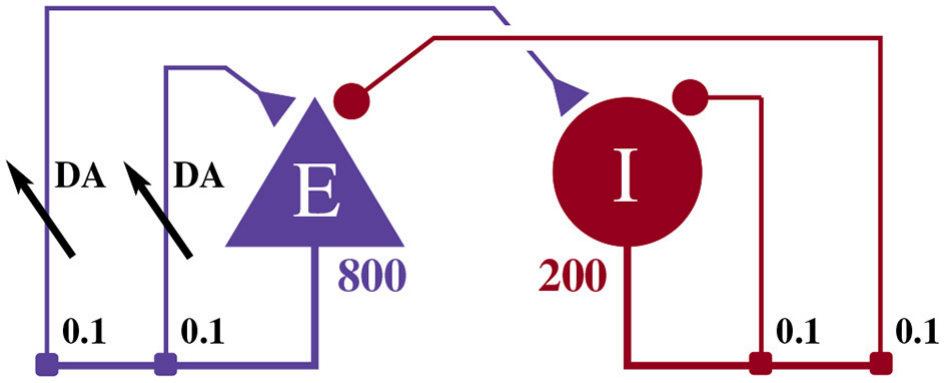
Illustration of the balanced random network model. The blue triangle represents the excitatory population, the red circle represents the inhibitory population, and the numbers beneath each symbol show the number of neurons in each population. Connection probabilities are shown in small bold numbers at the appropriate point in the connection matrix. All excitatory synaptic weights are plastic and initialized to 1 and all inhibitory synaptic weights are initialized to −1.

The model illustrated in Figure 2 consists of an 800 neuron excitatory population and a 200 neuron inhibitory population, within which, each neuron *i* is modeled using the Izhikevich model (Izhikevich, 2003) whose dimensionless membrane voltage *V*_*i*_ and adaption variables *U*_*i*_ evolve such that:

(4) $$\begin{array}{l} \frac{dV_{i}}{dt}=0.04V_{i}^{2}+5V_{i}+140-U_{i}+I_{{\text{syn}}_{i}}+I_{{\text{ext}}_{i}} \end{array}$$

(5) $$\begin{array}{l} \frac{dU_{i}}{dt}=a\left(bV_{i}-U_{i}\right) \end{array}$$

When the membrane voltage rises above 30, a spike is emitted and *V*_*i*_ is reset to *c* and *d* is added to *U*_*i*_. Excitatory neurons use the regular-spiking parameters (Izhikevich, 2003) where *a* = 0.02, *b* = 0.2, *c* = −65.0, *d* = 8.0 and inhibitory neurons use the fast-spiking parameters (Izhikevich, 2003) where *a* = 0.1, *b* = 0.2, *c* = −65.0, *d* = 2.0. Again, *I*_syn_*i*__ represents the synaptic input current and *I*_ext_*i*__ represents an external input current. While there are numerous ways to solve Equations (4) and (5) (Humphries and Gurney, 2007; Hopkins and Furber, 2015; Pauli et al., 2018), we chose to use the idiosyncratic forward Euler integration scheme employed by Izhikevich (2003) in the original work (Izhikevich, 2007). Under this scheme, Equation (4) is first integrated for two 0.5 ms timesteps and then, based on the updated value of *V*_*i*_, Equation (5) is integrated for a single 1 ms timestep.

#### 2.6.2. Synapse Models

The excitatory and inhibitory neural populations are connected recurrently, as shown in Figure 1, with instantaneous current-based synapses:

(6) $$\begin{array}{l} I_{{\text{syn}}_{i}}\left(t\right)=\overset{n}{\underset{j=0}{\sum }}w_{ij}\underset{t_{j}}{\sum }\delta \left(t-t_{j}\right), \end{array}$$

where *t*_*j*_ are the arrival times of incoming spikes from *n* presynaptic neurons. Inhibitory synapses are static with *w*_*ij*_ = −1.0 and excitatory synapses are plastic. Each plastic synapse has an eligibility trace *C*_*ij*_ as well as a synaptic weight *w*_*ij*_ and these evolve according to a three-factor STDP learning rule (Izhikevich, 2007):

(7) $$\begin{array}{l} \frac{dC_{ij}}{dt}=-\frac{C_{ij}}{\tau_{c}}+\text{STDP}\left(\Delta t\right)\delta \left(t-t_{\text{pre/post}}\right) \end{array}$$

(8) $$\begin{array}{l} \frac{dw_{ij}}{dt}=-C_{ij}D_{j} \end{array}$$

where τ_*c*_ = 1, 000*ms* represents the decay time constant of the eligibility trace and *STDP*(Δ*t*) describes the magnitude of changes made to the eligibility trace in response to the relative timing of a pair of pre and postsynaptic spikes with temporal difference Δ*t* = *t*_*post*_ − *t*_*pre*_. These changes are only applied to the trace at the times of pre and postsynaptic spikes as indicated by the Dirac delta function δ(*t* − *t*_pre/post_). Here, a double exponential STDP kernel is employed such that:

(9) $$\begin{array}{l} \text{STDP}\left(\Delta t\right)=\;\{\begin{array}{ll} A_{+}\exp\left(-\frac{\Delta t}{\tau_{+}}\right) & \text{if}\Delta t>0\\ A_{-}\exp\left(\frac{\Delta t}{\tau_{-}}\right) & \text{if}\Delta t<0\\ 0 & \text{otherwise} \end{array} \end{array}$$

where the time constants of the STDP window τ_+_ = τ_−_ = 20 ms and the strength of potentiation and depression are *A*_+_ = 0.1 and *A*_−_ = 0.15, respectively. Finally, each excitatory neuron has an additional variable *D*_*j*_ which describes extracellular dopamine concentration:

(10) $$\begin{array}{l} \frac{D_{j}}{t}=-\frac{D_{j}}{\tau_{d}}+\text{DA}\left(t\right) \end{array}$$

where τ_*d*_ = 200 ms represents the time constant of dopamine uptake and DA(*t*) the dopamine input over time.

#### 2.6.3. PyGeNN Implementation of Three-Factor STDP

The first step in implementing this learning rule in PyGeNN is to implement the STDP updates and decay of *C*_*ij*_ using GeNN's event-driven plasticity system, the implementation of which was described in our previous work (Knight and Nowotny, 2018). Using a similar syntax to that described in section 2.3, we first create a new “weight update model” with the learning rule parameters and the *w*_*ij*_ and *C*_*ij*_ state variables:


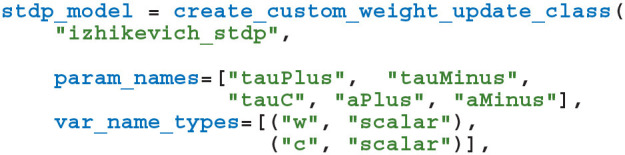


We then instruct GeNN to record the times of current and previous pre and postsynaptic spikes. The current spike time will equal the current time if a spike of this sort is being processed in the current timestep whereas the previous spike time only tracks spikes which have occurred *before* the current timestep:


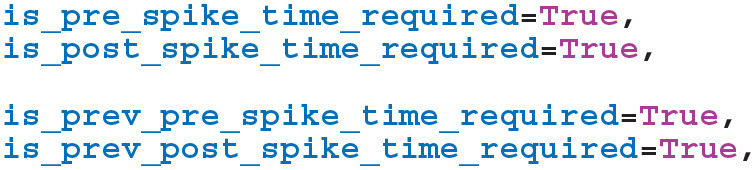


Next we define the “sim code” which is called whenever presynaptic spikes arrive at the synapse. This code first implements Equation (6)—adding the synaptic weight (*w*_*ij*_) to the postsynaptic neuron's input (*I*_syn_*i*__) using the $(addToInSyn,x) function.


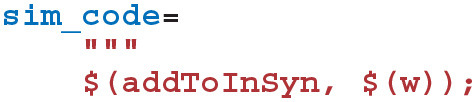


Within the sim code we also need to calculate the time that has elapsed since the last update of *C*_*ij*_ using the spike times we previously requested that GeNN record. Within a timestep, GeNN processes presynaptic spikes before postsynaptic spikes so the time of the last update to *C*_*ij*_ will be the latest time either type of spike was processed in previous timesteps:


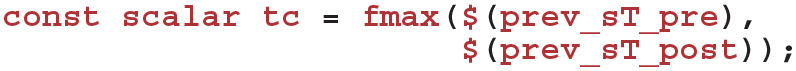


Using this time, we can now calculate how much to decay *C*_*ij*_ using the closed-form solution to Equation (7):


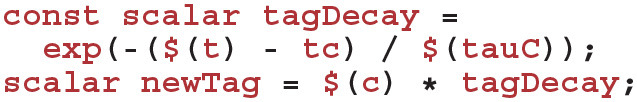


To complete the sim code we calculate the depression case of Equation (9) (here we use the *current* postsynaptic spike time as, if a postsynaptic and presynaptic spike occur in the same timestep, there should be no update).


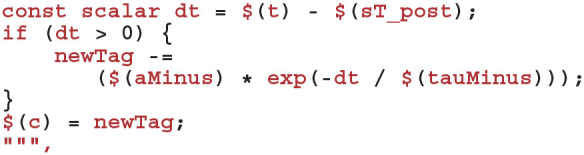


Finally, we define the “learn post code” which is called whenever a postsynaptic spike arrives at the synapse. Other than implementing the potentiation case of Equation (9) and using the *current* presynaptic spike time when calculating the time since the last update of *C*_*ij*_—in order to correctly handle presynaptic updates made in the same timestep—this code is very similar to the sim code:


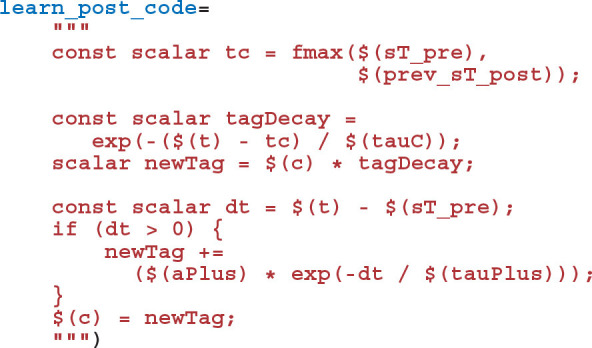


Adding the synaptic weight *w*_*ij*_ update described by Equation (8) requires two further additions to the model. As well as the pre and postsynaptic spikes, the weight update model needs to receive events whenever dopamine is injected via DA. GeNN supports such events via the “spike-like event” system which allows events to be triggered based on an expression evaluated on the presynaptic neuron. In this case, this expression simply tests an injectDopamine flag which gets set by the dopamine injection logic in our presynaptic neuron model:


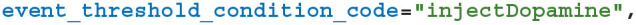


In order to extend our event-driven update of *C*_*ij*_ to include spike-like events we need to instruct GeNN to record the times at which they occur:


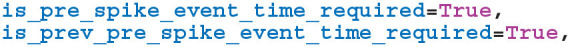


The spike-like events can now be handled using a final “event code” string:


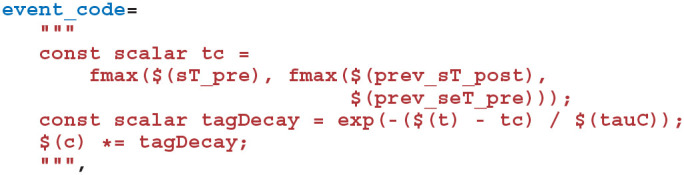


After updating the previously defined calculations of tc in the sim code and learn post code in the same way to also include the times of spike-like events, all that remains is to update *w*_*ij*_. Mikaitis et al. (2018) showed how Equation (8) could be solved algebraically, allowing *w*_*ij*_ to be updated in an event-driven manner with:

(11) $$\begin{array}{l} \Delta w_{ij}=\frac{C\left(t_{c}^{last}\right)D\left(t_{d}^{last}\right)}{-\left(\frac{1}{\tau_{c}}+\frac{1}{\tau_{d}}\right)}\left(e^{-\frac{t-t_{c}^{last}}{\tau_{c}}}e^{-\frac{t-t_{d}^{last}}{\tau_{d}}}-e^{-\frac{t_{w}^{last}-t_{c}^{last}}{\tau_{c}}}e^{-\frac{t_{w}^{last}-t_{d}^{last}}{\tau_{d}}}\right) \end{array}$$

where $t_{c}^{last}$, $t_{w}^{last}$, and $t_{d}^{last}$ represent the last times at which *C*_*ij*_, *W*_*ij*_, and *D*_*j*_, respectively were updated. Because we will always update *w*_*ij*_ and *C*_*ij*_ together when presynaptic, postsynaptic and spike-like events occur, $t_{c}^{last}=t_{w}^{last}$ and Equation (11) can be simplified to:

(12) $$\begin{array}{l} \Delta w_{ij}=\frac{C\left(t_{c}^{last}\right)D\left(t_{d}^{last}\right)}{-\left(\frac{1}{\tau_{c}}+\frac{1}{\tau_{d}}\right)}\left(e^{-\frac{t-t_{c}^{last}}{\tau_{c}}}e^{-\frac{t-t_{d}^{last}}{\tau_{d}}}-e^{-\frac{t_{c}^{last}-t_{d}^{last}}{\tau_{d}}}\right) \end{array}$$

and this update can now be added to each of our three event handling code strings to complete the implementation of the learning rule.

#### 2.6.4. PyGeNN Implementation of Pavlovian Conditioning Experiment

To perform the Pavlovian conditioning experiment described by Izhikevich (2007) using this model, we chose 100 random groups of 50 neurons (each representing stimuli *S*_1_…*S*_100_) from amongst the two neural populations. Stimuli are presented to the network in a random order, separated by intervals sampled from *U*(100, 300)ms. The neurons associated with an active stimulus are stimulated for a single 1 ms simulation timestep with a current of 40.0 nA, in addition to the random background current of *U*(−6.5, 6.5)nA, delivered to each neuron via *I*_ext_*i*__ throughout the simulation. *S*_1_ is arbitrarily chosen as the Conditioned Stimuli (CS) and, whenever this stimuli is presented, a reward in the form of an increase in dopamine is delivered by setting DA(*t*) = 0.5 after a delay sampled from *U*(0, 1000)ms. This delay period is large enough to allow a few irrelevant stimuli to be presented which act as distractors. The simplest way to implement this stimulation regime is to add a current source to the excitatory and inhibitory neuron populations which adds the uniformly-distributed input current to an externally-controllable per-neuron current. In PyGeNN, the following model can be defined to do just that:


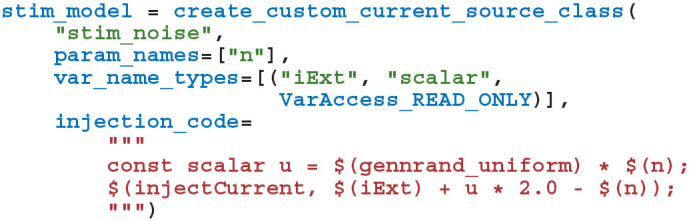


where the n parameter sets the magnitude of the background noise, the $(injectCurrent, I) function injects a current of *I*nA into the neuron and $(gennrand_uniform) samples from *U*(0, 1) using the “XORWOW” pseudo-random number generator provided by cuRAND (NVIDIA Corporation, 2019). Once a current source population using this model has been instantiated and a memory view to iExt obtained in the manner described in section 2.3, in timesteps when stimulus injection is required, current can be injected into the list of neurons contained in stimuli_input_set with:


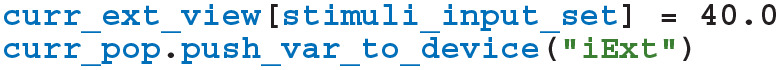


The same approach can then be used to zero the current afterwards.

## 3. Results

In the following subsections we will analyse the performance of the models introduced in sections 2.5 and 2.6 on a representative selection of NVIDIA GPU hardware:

- Jetson Xavier NX—a low-power embedded system with a GPU based on the Volta architecture with 8 GB of shared memory.
- GeForce GTX 1050Ti—a low-end desktop GPU based on the Pascal architecture with 4 GB of dedicated memory.
- GeForce GTX 1650—a low-end desktop GPU based on the Turing architecture with 4 GB of dedicated memory.
- Titan RTX—a high-end workstation GPU based on the Turing architecture with 24 GB of dedicated memory.

All of these systems run Ubuntu 18 apart from the system with the GeForce 1050 Ti which runs Windows 10.

### 3.1. Cortical Microcircuit Model Performance

**Figure 3:**
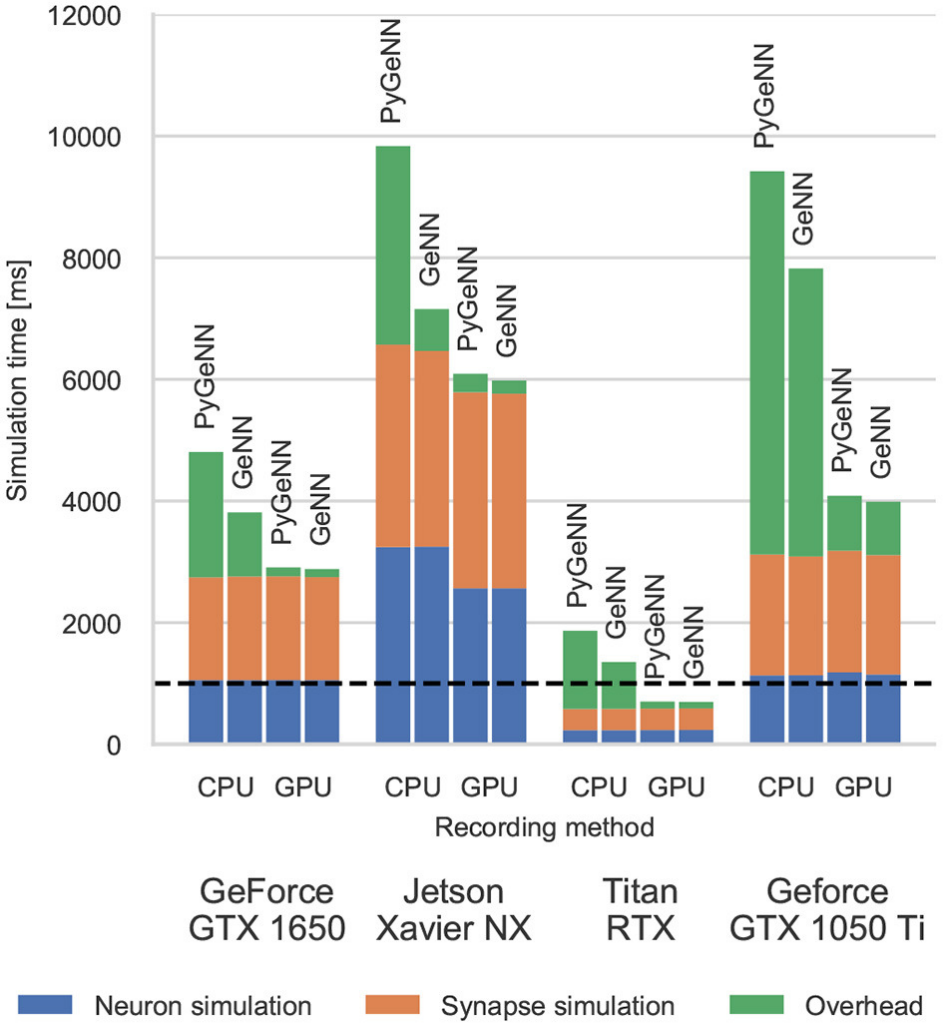
Simulation times of the microcircuit model running on various GPU hardware for 1 s of biological time. “Overhead” refers to time spent in simulation loop but not within CUDA kernels. The dashed horizontal line indicates realtime performance.

Figure 3 shows the simulation times for the full-scale microcircuit model. We measured the total simulation time by querying the std::chrono::high_resolution_clock in C++ and the time.perf_counter in Python before and after the simulation loop; and used CUDA's own event timing system (NVIDIA Corporation, 2021, Section 3.2.6.7) to record the time taken by the neuron and synapse kernels. As one might predict, the Jetson Xavier NX is slower than the three desktop GPUs but, considering that it only consumes a maximum of 15 W compared to 75 or 320 W for the GeForce cards and Titan RTX, respectively, it still performs impressively. The time taken to actually simulate the models (“Neuron simulation” and “Synapse simulation”) are the same when using PyGeNN and GeNN as all optimisation options are exposed to PyGeNN. Interestingly, when simulating *this* model, the larger L1 cache and architectural improvements present in the Turing-based GTX 1650 do not result in significantly improved performance over the Pascal-based GTX 1050Ti. Instead, the slightly improved performance of the GTX 1650 can probably be explained by its additional 128 CUDA cores.

Without the recording system described in section 2.4, the CPU and GPU need to be synchronized after every timestep to allow spike data to be copied off the GPU and stored in a suitable data structure. The “overheads” shown in Figure 3 indicate the time taken by these processes as well as the unavoidable overheads of launching CUDA kernels etc. Because Python is an interpreted language, updating the spike data structures is somewhat slower and this is particularly noticeable on devices with a slower CPU such as the Jetson Xavier NX. However, unlike the desktop GPUs, the Jetson Xavier NX's 8 GB of memory is shared between the GPU and the CPU meaning that data does not need to be copied between their memories and can instead by accessed by both. While, using this shared memory for recording spikes reduces the overhead of copying data off the device, because the GPU and CPU caches are not coherent, caching must be disabled on this memory which reduces the performance of the neuron kernel. Although the Windows machine has a relatively powerful CPU, the overheads measured in both the PyGeNN and GeNN simulations run on this system are extremely large due to additional queuing between the application and the GPU driver caused by the Windows Display Driver Model (WDDM). When small—in this case 0.1 ms—simulation timesteps are used, this makes per-timestep synchronization disproportionately expensive.

However, when the spike recording system described in section 2.4 is used, spike data is kept in GPU memory until the end of the simulation and overheads are reduced by up to 10×. Because synchronization with the CPU is no longer required every timestep, simulations run approximately twice as fast on the Windows machine. Furthermore, on the high-end desktop GPU, the simulation now runs faster than real-time in both PyGeNN and GeNN versions—significantly faster than other recently published GPU simulators (Golosio et al., 2021) and even specialized neuromorphic systems (Rhodes et al., 2020).

### 3.2. Pavlovian Conditioning Performance

**Figure 4:**
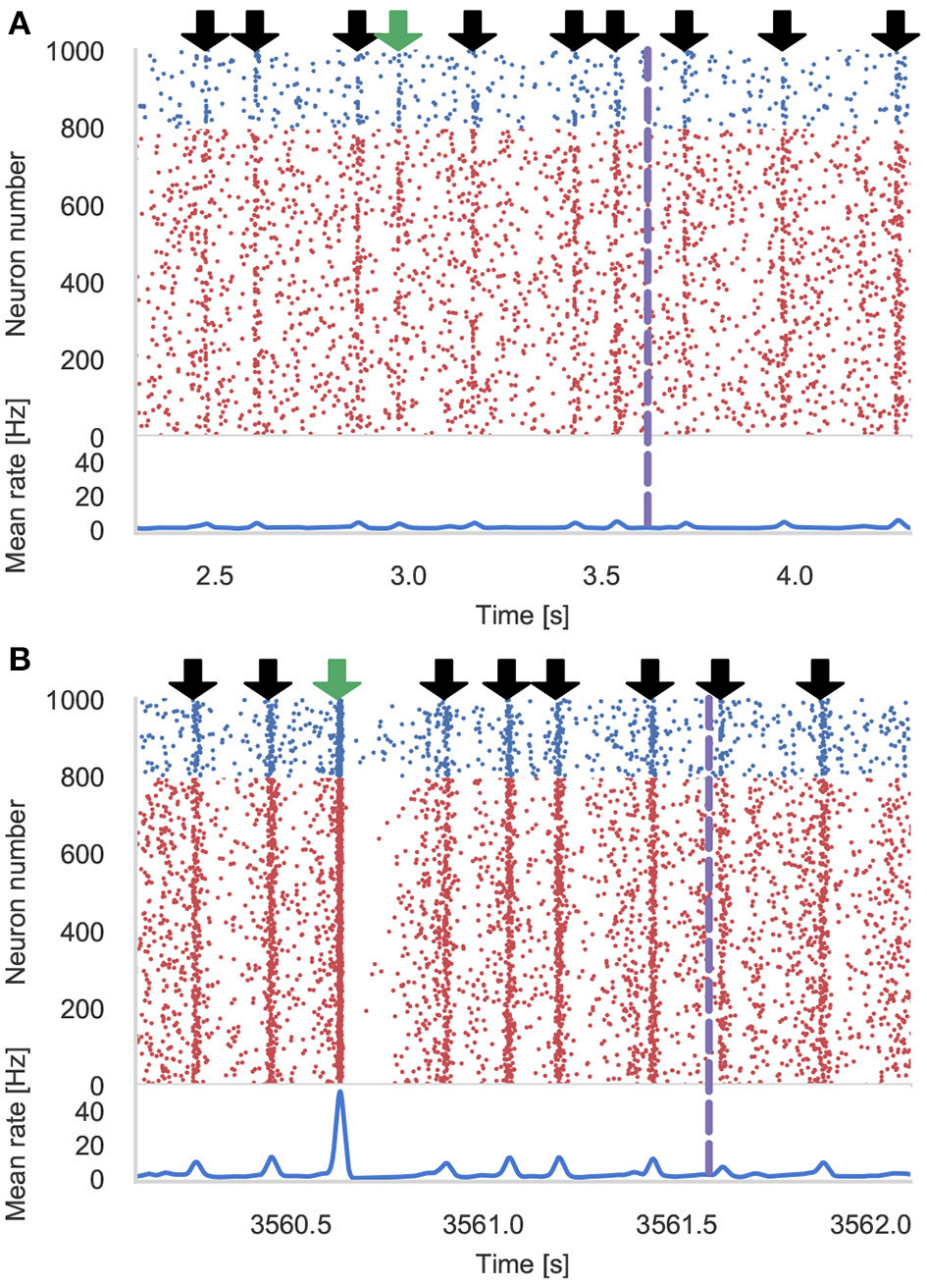
Results of Pavlovian conditioning experiment. Raster plot and spike density function (SDF) (Szücs, 1998) showing the activity centered around the first delivery of the Conditioned Stimulus (CS) during initial **(A)** and final **(B)** 50 s of simulation. Downward green arrows indicate times at which the CS is delivered and downward black arrows indicate times when other, un-rewarded stimuli are delivered. Vertical dashed lines indicate times at which dopamine is delivered. The population SDF was calculated by convolving the spikes with a Gaussian kernel of σ = 10 ms width.

**Figure 5:**
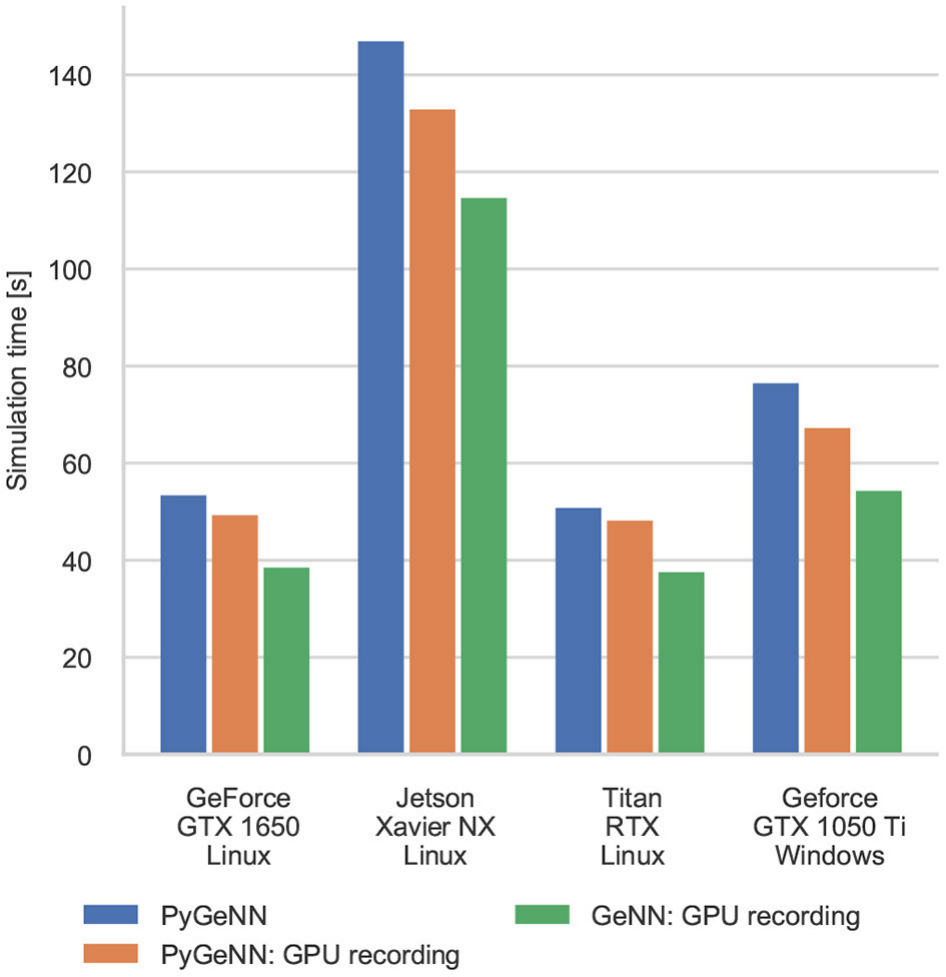
Simulation times of the Pavlovian Conditioning model running on various GPU hardware for 1 h of biological time. “GPU recording” indicates simulations where the new recording system is employed. Times are taken from averages calculated over 5 runs of each model.

Figure 4 shows the results of an example simulation of the Pavlovian conditioning model. At the beginning of each simulation (Figure 4A), the neurons representing every stimulus respond equally. However, after 1 h of simulation, the response to the CS becomes much stronger (Figure 4B)—showing that these neurons have been selectively associated with the stimulus even in the presence of the distractors and the delayed reward. In Figure 5, we show the runtime performance of simulations of the Pavlovian conditioning model, running on the GPUs described above using PyGeNN with and without the recording system described in section 2.4. These PyGeNN results are compared to a GeNN simulation which also uses the recording system. Because each simulation timestep only takes a few μs, the overhead of using CUDA timing events significantly alters the performance so, for this model, we only measure the duration of the simulation loop using the approaches described in the previous section. Although we only record the spiking activity during the first and last 50 s, using the recording system still significantly improves the overall performance on all devices—especially on the Jetson Xavier NX with its slower CPU. Interestingly the Titan RTX and GTX 1650 perform identically in this benchmark with speedups ranging from 62× to 72× real-time. This is because, as discussed previously, this model is simply not large enough to fill the 4,608 CUDA cores present on the Titan RTX. Therefore, as the two GPUs share the same Turing architecture and have very similar clock speeds (1,350–1,770 MHz for the Titan RTX and 1,485–1,665 MHz for the GTX 1650), the two GPUs perform very similarly. As for the simulations of the microcircuit model, the Jetson Xavier NX performs rather slower than the desktop GPUs but still achieves speedups of up to 31×.

Interestingly, unlike in the simulations of the microcircuit model, here the GTX 1050 Ti performs rather differently. Although the clock speed of this device is approximately the same as the other GPUs (1,290–1,392 MHz) and it has a similar number of CUDA cores to the GTX 1650, its performance is significantly worse. The difference in performance across all configurations is likely to be due to architectural differences between the older Pascal; and newer Volta and Turing architectures. Specifically, Pascal GPUs have one type of Arithmetic Logic Unit (ALU) which handles both integer and floating point arithmetic, whereas the newer Volta and Turing architectures have equal numbers of dedicated integer and floating point ALUs as well as significantly larger L1 caches. As discussed in our previous work (Knight and Nowotny, 2018), these architectural features are particularly beneficial for SNN simulations with STDP where a large amount of floating point computation is required to update the synaptic state *and* additional integer arithmetic is required to calculate the indices into the sparse matrix data structures.

**Figure 6:**
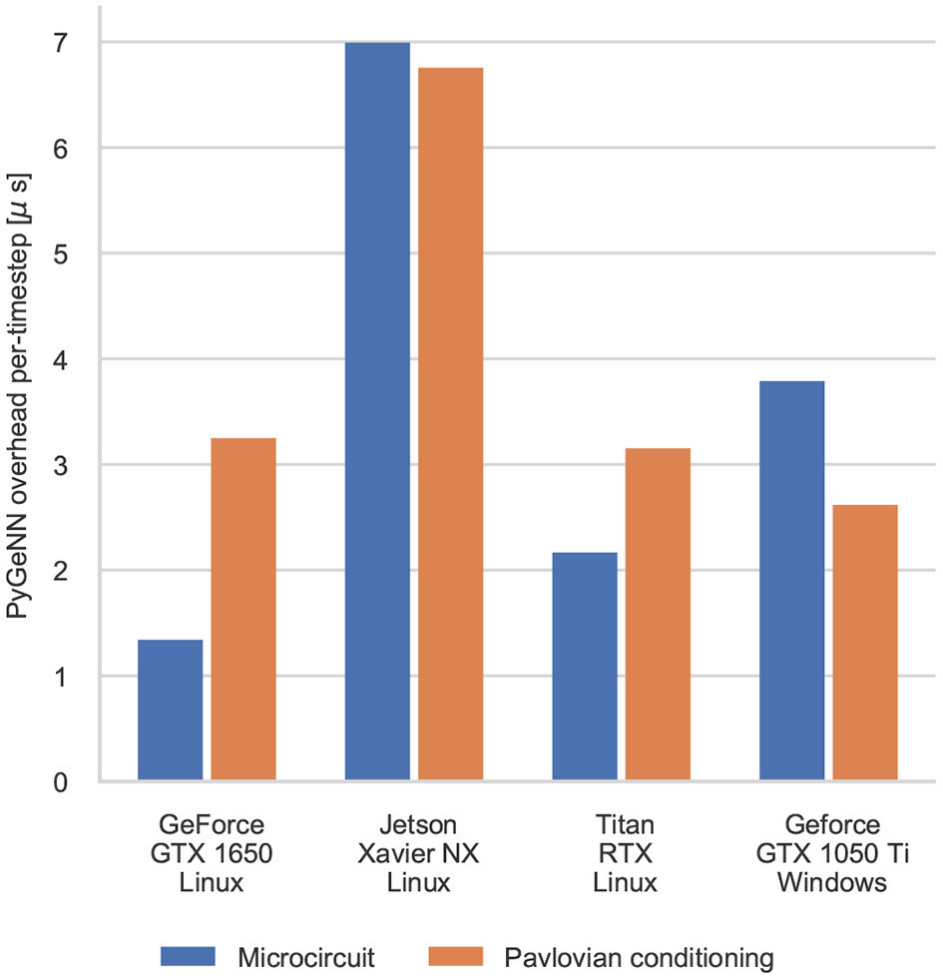
Comparison of the duration of individual timestep in PyGeNN and GeNN simulations of the microcircuit and Pavlovian conditioning experiments. Times are taken from averages calculated over 5 runs using the GPU recording system.

The difference between the speeds of the PyGeNN and GeNN simulations of the Pavlovian conditioning model (Figure 5) *appear* much larger than those of the microcircuit model (Figure 3). However, as Figure 6 illustrates, for individual timesteps the excess time due to overheads is approximately the same for both models and consistent with the cost of a small number of Python to C++ function calls (Apache Crail, 2019). Depending on the size and complexity of the model as well as the hardware used, this overhead may or may not be important. For example, when simulating the microcircuit model for 1 s on the Titan RTX, the overhead of using PyGeNN is <0.2 % but, when simulating the Pavlovian conditioning model on the same device, the overhead of using PyGeNN is almost 31 %.

## 4. Discussion

In this paper we have introduced PyGeNN, a Python interface to the C++ based GeNN library for GPU accelerated spiking neural network simulations.

Uniquely, the new interface provides access to all the features of GeNN, without leaving the comparative simplicity of Python and with, as we have shown, typically negligible overheads from the Python bindings. PyGeNN also allows bespoke neuron and synapse models to be defined from within Python, making PyGeNN much more flexible and broadly applicable than, for instance, the Python interface to NEST (Eppler et al., 2009) or the PyNN model description language used to expose CARLsim to Python (Balaji et al., 2020).

In many ways, the new interface resembles elements of the Python-based Brian 2 simulator (Stimberg et al., 2019) (and it's Brian2GeNN backend; Stimberg et al., 2020) with two key differences. Unlike in Brian 2, bespoke models in PyGeNN are defined with “C-like” code snippets. This has the advantage of unparalleled flexibility for the expert user, but comes at the cost of more complexity as the code for a timestep update needs to include a suitable solver and not merely differential equations. The second difference lies in how data structures are handled. Whereas simulations run using the C++ or Brian2GeNN Brian 2 backends use files to exchange data with Python, the underlying GeNN data structures are directly accessible from PyGeNN meaning that no disk access is involved.

As we have demonstrated, the PyGeNN wrapper, exactly like native GeNN, can be used on a variety of hardware from data center scale down to mobile devices such as the NVIDIA Jetson. This allows for the same codes to be used in large-scale brain simulations and embedded and embodied spiking neural network research. Supporting the popular Python language in this interface makes this ecosystem available to a wider audience of researchers in both Computational Neuroscience, bio-mimetic machine learning and autonomous robotics.

The new interface also opens up opportunities to support researchers that work with other Python based systems. In the Computational Neuroscience and Neuromorphic computing communities, we can now build a PyNN (Davison et al., 2008) interface on top of PyGeNN and, in fact, a prototype of such an interface is in development. Furthermore, for the burgeoning spike-based machine learning community, we can use PyGeNN as the basis for a spike-based machine learning framework akin to TensorFlow or PyTorch for rate-based models. A prototype interface of this sort called mlGeNN is in development and close to release.

In this work we have introduced a new spike recording system for GeNN and have shown that, using this system, we can now simulate the Potjans microcircuit model (Potjans and Diesmann, 2014) faster than real-time and, to the best of our knowledge, faster than any other system. Finally, the excellent performance we have demonstrated using low-end Turing architecture GPUs is very exciting in terms of increasing the accessibility of GPU accelerated Computational Neuroscience and SNN machine learning research.

## Data Availability Statement

All models, data and analysis scripts used for this study can be found in https://github.com/BrainsOnBoard/pygenn_paper. All experiments were carried out using the GeNN 4.4.0 which is fully open source and available from https://doi.org/10.5281/zenodo.4419159.

## Author Contributions

JK and TN wrote the paper. TN was the original developer of GeNN. AK is the original developer of PyGeNN. JK is currently the primary developer of both GeNN and PyGeNN, responsible for implementing the spike recording system, and performed the experiments and the analysis of the results that are presented in this work. All authors contributed to the article and approved the submitted version.

## Conflict of Interest

The authors declare that the research was conducted in the absence of any commercial or financial relationships that could be construed as a potential conflict of interest.
